# *Citrus bergamia* Extract, a Natural Approach for Cholesterol and Lipid Metabolism Management: A Randomized, Double-Blind Placebo-Controlled Clinical Trial

**DOI:** 10.3390/foods13233883

**Published:** 2024-11-30

**Authors:** Amelia Spina, Fabio Amone, Vincenzo Zaccaria, Violetta Insolia, Anna Perri, Danilo Lofaro, Francesco Puoci, Vincenzo Nobile

**Affiliations:** 1 Nutratech S.r.l., Spin-Off of University of Calabria, 87036 Rende, Italy; amelia.spina@nutratechtesting.com (A.S.); fabio.amone@nutratechtesting.com (F.A.); 2 R&D Department, Bionap S.r.l., 95032 Belpasso, Italy; v.zaccaria@bionap.com; 3 Alma Mater Europea, 2000 Maribor, Slovenia; violetta.insolia@almamater.si; 4 Department of Experimental and Clinical Medicine, Magna Grecia University of Catanzaro, 88100 Catanzaro, Italy; 5 Department of Mechanical, Energy, Management Engineering, University of Calabria, 87036 Rende, Italy; 6 Department of Pharmacy, Health and Nutritional Sciences, University of Calabria, 87100 Cosenza, Italy; 7 R&D Department, Complife Italia S.r.l., 27028 San Martino Siccomario, Italy

**Keywords:** flavonoids, cholesterol, lipid management, cardiovascular disease, clinical trial

## Abstract

Strategies for controlling cholesterol and lipid metabolism, including the use of food supplements, are part of the non-pharmacological intervention to ameliorate cardiovascular health. To demonstrate the efficacy of a standardized flavonoids (150 mg/day) extract from *Citrus bergamia* on cholesterol and lipid management, a placebo-controlled clinical trial on 64 subjects with high cholesterol was carried out. The total study duration was 4 months, with intermediate checkpoints at 1-month intervals. Low-density lipoprotein (LDL-C), high-density lipoprotein (HDL-C) cholesterol, total cholesterol (TC) levels, oxidized low-density lipoprotein (ox-LDL), and paraoxonase activity (PON1) were measured as primary endpoints (efficacy evaluation), while weight, blood pressure, hepatic and renal function blood markers were measured as secondary endpoints (safety evaluation). After 4 months, both TC and LDL-C significantly decreased by 8.8% and 11.5%, respectively, along with a 5.5% increase in HDL-C which was trending towards significance. In addition, ox-LDL was significantly reduced by 2.0%, while PON1 was significantly increased by 6.5%. The secondary endpoints were not changed during the study, demonstrating a good tolerability of the test product. Our findings demonstrate the efficacy of the extract as a natural approach for cholesterol and lipid metabolism management.

## 1. Introduction

Hypercholesterolemia is an asymptomatic condition with an estimated global prevalence of 39% [1]. Raised cholesterol levels are a well-recognized risk factor for cardiovascular (CVD) and cerebrovascular diseases, causing an estimated 2.6 million (4.5% of global) deaths and 29.7 million Disability Adjusted Life Years (DALYS) [1,2].

Another condition characterized by hypercholesterolemia and high triglycerides levels is the Metabolic Syndrome (MetS), defined as a cluster of metabolic abnormalities including impairments in lipid, glucose, carbohydrates, and protein metabolism [3].

The main therapeutic approach for hypercholesterolemia treatment is based on statins [4,5,6,7,8]. Statins belong to 3-hydroxy-3-methyl-glutaryl-coenzyme A (HMG-CoA) reductase inhibitors and have been shown to effectively reduce the low-density lipoprotein cholesterol (LDL-C) [8,9,10,11,12]. Despite their good global safety profile, statins are associated with numerous adverse effects, including musculoskeletal pain, headache, respiratory infections, gastrointestinal events, increased liver transaminases, and increased blood glucose and glycated hemoglobin (HbA1c) levels [4,13,14]. Among these, the statin-associated muscle symptoms (SAMS) [15] are the main cause of statin therapy discontinuation. In borderline conditions, food supplements can be used to support the physiology of the body to improve the lipid metabolism by decreasing the total cholesterol, the associated LDL and triglycerides (TGs) levels, and by increasing the high-density lipoprotein cholesterol (HDL-C) or supporting the liver function. Both the market and the scientific communities are exploring and developing alternative and complementary approaches with fewer complications to help the body maintain normal cholesterol levels and, in the meantime, decrease the risk of development of metabolic illness and CVDs [16].

The efficacy of nutraceuticals in improving dyslipidemia is well-known [17,18,19,20,21], even if some trials have yielded conflicting results [22,23,24]. In recent years, the importance of nutraceuticals in optimizing the lipid-lowering efficacy in subjects with low-to-moderate hypercholesterolemia and in subjects with statin-associated side effects has been recognized by the International Expert Lipid Panel (ILEP) [25,26]. The intake of various nutraceuticals, including phytosterols/stanols and botanical extracts containing polyphenols, is also recommended by the European Society of Cardiology (ESC) as part of normal lifestyle interventions to reduce the total cholesterol (TC) and LDL-C levels or in subjects who do not qualify for treatment with statins [27]. Polyphenols are key mediators of the beneficial effects of lipid-lowering potential that has been widely described in literature and could be associated with different mechanisms such as inhibition or reduction of intestinal transport of cholesterol and TGs; reduction of serum cholesterol, TGs, and lipoproteins; and interaction with cholesterol and TG synthesis and body clearance. Among the most well-known and researched polyphenols involved in these activities are flavonoids, followed by stilbenes (i.e., resveratrol), phenolic acids, and lignans [28].

The lipid-lowering effects of bergamot flavonoids can be attributed to their multifaceted interaction with lipid metabolism pathways [29]. These flavonoids activate sirtuin-1 and AMPK-α, central regulators of cellular energy homeostasis, which promote fatty acid oxidation through carnitine palmitoyltransferase 1 (CPT1) activation [30,31,32,33], and concurrently reduce very-low-density lipoprotein (VLDL) synthesis by inhibiting hepatocyte nuclear factor 4 (HNF4) [34] and sterol regulatory element-binding protein 1 (SREBP-1) [35]. Additionally, bergamot flavonoids enhance LDL receptor activity by two distinct mechanisms: increasing gene transcription via protein kinase C (PKC) activation [36] and facilitating receptor translocation to the plasma membrane through peroxisome proliferator-activated receptor gamma (PPAR-γ) activation [37]. These molecular actions contribute to a significant reduction in circulating LDL-C levels and a favorable shift from small, dense LDL particles to larger, less atherogenic subfractions, thereby improving the overall lipid profile.

In this study we aimed to assess the efficacy of a bergamot extract standardized in flavonoids in reducing the levels of LDL-C and improving the lipidic profile. Bergamot is a citrus fruit derived from *Citrus bergamia* and is native to southern Italy, where it is cultivated along the coastal strip, especially widespread in the Calabria region. The phytochemicals found in bergamot extract includes flavanones, flavonoids, flavones O-glucosides, and C-glucosides [38]. Among all the others citrus fruits, the bergamot contains an especially high content of flavonoids [39,40,41,42].

The effect of the bergamot polyphenol fraction in lowering LDL-C and TC in humans has been shown by different studies [43,44,45,46,47]. However, its effects in increasing high-density lipoprotein cholesterol (HDL-C) and reducing triglycerides (TG) are inconsistent across all studies [38,48]. This inconsistency can be explained by the methods of extraction, preparation, and standardization that was different from one trial to another. In addition, some trials used an open-label design [38] or a 6-month prospective trial without placebo control [29]. The present study aimed to add an additional piece of information on the efficacy of the bergamot in improving the dyslipidemia in the short and long term in subjects with borderline-high LDL-C levels, while linking it with a standardized and reproducible amount and profile of flavonoids.

## 2. Materials and Methods

### 2.1. Trial Design

This was a single-center, randomized, balanced-randomization, placebo-controlled, parallel-group trial conducted in Italy at the Nutratech S.r.l. facilities (Rende, CS, Italy) between April 2022 and May 2023. It consisted of a screening visit, a baseline visit (M0), and 3 follow-up visits (from M2 = month 2 to M4 = month 4) over a 4 month supplementation period. The enrollment of eligible subjects was based on the results of the blood analysis of low-density lipoprotein (LDL) levels. After the enrollment subjects were randomly assigned to the active or placebo group (1:1 allocation rate) at baseline. All the study endpoints were measured at each checkpoint.

The trial protocol (H.E.HU.AC.NMS00.210.00.00_IT0002159/22) was approved on 21 March 2022 by the Ethics Committee of the University of Calabria. The trial was registered at ISCRTN registry, number ISRCTN90859063 (https://doi.org/10.1186/ISRCTN90859063, accessed on 18 October 2024).

During the screening visit, subjects were informed about the trial’s risks, benefits, and procedures and signed an informed consent form before participating in the trial. The ethical principles of the Helsinki declaration were applied to all the study procedures.

### 2.2. Participants

Eligible participants were all healthy men and women aged between 40 and 70 years old with untreated dyslipidemia. Before inclusion in the trials, the eligible participants underwent a blood test to evaluate their LDL levels. Only subjects with LDL levels between 160 and 190 mg/dL and body mass index (BMI) between 25 and 29.9 were enrolled in the trial. Exclusion criteria included statin therapy or nutraceutical use one month before the study start; vegetarianism; changes in routinary lifestyle (including the physical activity and alimentary habits within 2 weeks before the screening visit); positive anamnesis for pathologies having relevant effects on the gastrointestinal system or the visceral motility; pregnancy or breastfeeding (only for women); smoking; a positive medical history of pathologies or pharmacological treatment that could potentially interfere with the test product; discontinuation of the test product intake for more than one week (low compliance with treatment); planning a diet or using weight control products; history of drug, alcohol, or substance abuse; known food intolerance or food allergies; participation in a similar trial within the previous month; unstable medical pathologies (e.g., cardiac arrhythmias or ischemia, uncontrolled hypertension or hypotension, kidney failure, and diabetes mellitus); history of paralysis or cerebrovascular accident; active cancers undergoing chemotherapy; use of diuretics one months before the screening visit; and any condition that limits the subject’s ability to cooperate during the study.

### 2.3. Interventions and Randomization

The active treatment arm received one capsule daily containing 375 mg of a commercially available bergamot (*Citrus bergamia*) extract (Bergavit^TM^, Bionap S.r.l., Piano Tavola Belpasso, CT, Italy), 134 mg pregelatinized starch, 0.4 mg magnesium stearate, 0.3 mg talc, and 0.3 mg silica. The placebo group was administered one capsule daily, identical in appearance to the active capsule, but containing the same ingredients, with the exception of bergamot extract, which was substituted with 375 mg maltodextrin. The total intake of standardized flavonoids from bergamot in the active group was 150 mg/day (including neohesperidin, naringin, and neoeriocitrin).

Participants were assigned to receive either the active or the placebo product based on a computer-generated randomization list (PASS 11, version 11.0.8, PASS, LLC, Kaysville, UT, USA), with a restricted and balanced 1:1 ratio. The randomization was performed using the “Efron’s biased coin” algorithm. The active and placebo products were identical in size and shape and were numbered. The randomization list was kept confidential in sequentially numbered, sealed, and opaque envelopes. Neither the investigator nor the participant knew the product assignment.

### 2.4. Clinical Biochemistry

Blood samples were drawn after an overnight fast (~12 h) by a nurse in a clinical biochemistry laboratory. Low-density lipoprotein (LDL-C), high-density lipoprotein (HDL-C) cholesterol, total cholesterol (TC), oxidized low-density lipoprotein (ox-LDL), and paraoxonase activity (PON1) were the primary endpoints of the study. The secondary endpoints included the measurement of weight, blood pressure, triglycerides (TG), aspartate aminotransferase (AST), alanine aminotransferase (ALT), gamma-glutamyl transferase (GGT), glycated hemoglobin (HbA1c), paraoxonase activity (PON1), C-reactive protein (CRP), and creatine (CR). All the hematic parameters were measured using routine laboratory methods. The HDL-C concentration was calculated using the Friedewald formula [49].

### 2.5. Stastistical Methods

Pairwise comparison of the data were conducted using NCSS 10 (v10.0.7 for Windows, NCSS, Kaysville, UT, USA). The significance levels were reported as follows: * *p* < 0.05, ** *p* < 0.01, and *** *p* < 0.001.

## 3. Results

### 3.1. Subject Characteristics

**Figure 1:**
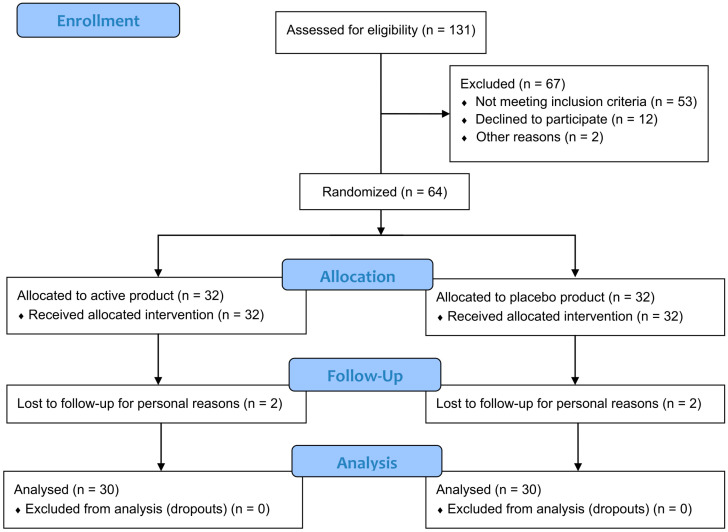
Participants flow diagram.

The study screened one hundred and thirty-one subjects (n = 131), 67 of which were excluded for the following reasons: 53 did not meet the inclusion criteria (mainly related to LDL-C and BMI), 12 declined to participate, and two decided to start a statin therapy. The study successfully randomized 64 subjects with high LDL-C levels; 32 subjects were randomized to the active treatment arm, and 32 to the placebo treatment arm. The per-protocol (PP) population comprised sixty subjects (n = 60), with thirty subjects (n = 30) in each treatment group. The reason for being excluded from the PP population (2 subjects in each treatment group) was due to personal reasons not related to products use. The Bergavit^TM^ trial participants flow chart is reported in Figure 1.

The mean age was 50.9 ± 1.7 in the active group and 51.5 ± 1.6 in the placebo group. In both groups, the LDL-C levels were in the range 160 and 190 mg/dL, while the TC levels were approximately 20% (borderline high category) above the threshold (normal) value (200 mg/dL). Additional demographics and baseline data are reported in Table 1.

### 3.2. Primary Endpoints

**Figure 2:**
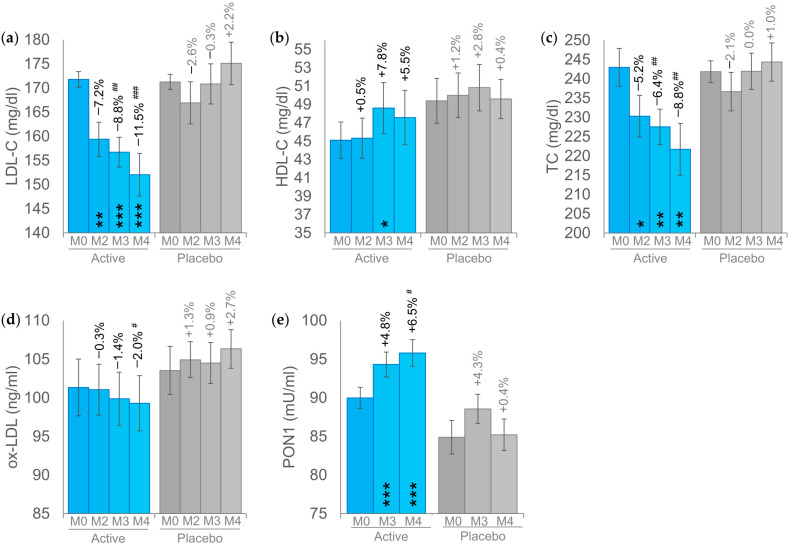
Results of the primary endpoints. (**a**) Low-density lipoprotein cholesterol (LDL-C) levels. (**b**) High-density lipoprotein (HDL-C) cholesterol levels. (**c**) Total cholesterol (TC) levels. (**d**) Oxidized low-density lipoprotein (ox-LDL). (**e**) Paraoxonase activity (PON1). The intragroup (vs. baseline) statistical analysis is reported within the bars by the symbol *, while the intergroup (active vs. placebo) statistical analysis is reported above the bars by the symbol #, as follows: */# *p* < 0.05, **/## *p* < 0.01, ***/### *p* < 0.001.

During the screening visit, the LDL-C blood levels was used to enroll subjects with high LDL-C levels (between 160 and 190 mg/dL). The LDL-C blood levels at baseline were 171.8 ± 1.6 in the active group and 171.3 ± 1.5 in the placebo group. In the active group, the LDL-C levels were reduced after the first 2 months of treatment (159.4 ± 3.5, *p* < 0.01) by −7.2% and further reduced by −8.8% (156.7 ± 3.1, *p* < 0.001) and 11.5% (152.0 ± 4.4, *p* < 0.001) after 3 and 4 months of intake, respectively (Figure 2a). Interestingly, the LDL-C blood levels after 2 months of product intake was classified in the “high” range to the “borderline high” range. The LDL-C blood levels in the placebo group was increased by a non-significant 2.2% (*p* > 0.05). The variation of LDL-C between the active and the placebo product was statistically different at M3 and M4.

The product’s effect on HDL-C was not as clear as its effect on LDL-C (Figure 2b). The HDL-C was statistically significantly increased by +7.8% (48.6 ± 2.8, *p* < 0.05) after three months of product intake, while the increase (+5.5%) after four months of product intake was statistically borderline (*p* = 0.067). The variation of HDL-C was not statistically significant in the placebo group as well as the variation between the active and placebo product.

The intake of the active product determined a reduction of the TC by 5.2% (230.3 ± 5.4, *p* < 0.05), 6.4% (227.5 ± 4.6, *p* < 0.01), and 8.8% (221.7 ± 6.7, *p* < 0.01), after two, three, and four months of product intake, respectively (Figure 2c). The product effect in reducing the TC was statistically significant starting from M3 (*p* < 0.01) and continued at M4 (*p* < 0.01). Interestingly, the classification of TC levels decreased from “high” at baseline (243.0 ± 4.9) to “moderately high” (from 200 to 239 mg/dL) starting from M2. TC blood levels in the placebo group remained unchanged (*p* > 0.05).

A decreasing trend was observed for the ox-LDL (Figure 2d). This trend was not statistically significant over the time of treatment even if a statistically significant difference between the active (−2.0%) and the placebo product (+2.7%) was seen at M4. Ox-LDL remained unchanged (*p* > 0.05) in the placebo group.

PON1 activity was statistically significantly increased in the active group by +4.8% (*p* < 0.001) and 6.5% (*p* < 0.001) after three and four months of treatment, respectively (Figure 2d). The difference between the active and the placebo product was seen at M4 (*p* < 0.05). PON1 activity was not influenced by the placebo treatment.

### 3.3. Secondary Endpoints

Both the active and placebo products were well-tolerated. All the markers of hepatic (AST, ALT, GGT and CRP) and renal function (CR) were not changed. Interestingly, the glucose blood concentration and the HbA1c were also not influenced by the treatment (Table 2). Interestingly, we observed, at M4, a statistically significant decrease (*p* < 0.01) of the systolic blood pressure by 7.0% (8.6 mmHg). This variation was statistically borderline (*p* = 0.05) compared to the placebo. Triglycerides were progressively reduced at all check points up to 16.7% in the active group at M4 compared to 7.4% in the placebo group, although the differences were not statistically significant.

## 4. Discussion

The use of food supplements for cholesterol and lipid metabolism management is growing rapidly and steadily in European countries [50,51,52]. Non-pharmacological interventions, and especially naturally-derived ingredients, are well-accepted by consumers due to a better perception of their toxicological and environmentally-friendly profile compared to drugs [53].

Lowering LDL-C is a well-recognized factor to reduce the risk of major vascular events [54,55] and a key target in the prevention of atherosclerotic cardiovascular disease (ASCVD) [56]. Clinical guidelines from medical associations recommend lifestyle changes, the use of food supplement, or, when indicated, pharmacological interventions to lower LDL-C and to decrease ASCVD risk [27,57,58]. Given the limited impact of lifestyle changes on cholesterol improvement, the low long-term adherence of patients to these changes, and the necessity of achieving low LDL-C levels, there is increasing interest in natural compounds that can safely enhance lipid profiles. Several lipid-lowering nutraceuticals have been identified and clinically tested, although their effectiveness and tolerability [51,52,54,56].

The beneficial role of bergamot in lowering LDL-C is well-documented in the literature. Beyond its recognized effects, bergamot flavonoids exert their lipid-lowering action through multifaceted mechanisms, including the modulation of lipid metabolism pathways and enhanced clearance of LDL particles [29]. However, as highlighted in a meta-analysis, these findings are based on studies with relatively small sample sizes, underscoring the need for further research to strengthen the causal relationship between bergamot extract consumption and improvements in lipid profiles [2]. In this context, our study aimed to evaluate the efficacy of a natural and standardized (reproducible) extract obtained from bergamot juice in improving the dyslipidemia in both the short and long term in subjects with high LDL-C levels. The extract composition was standardized and contained 150 mg of flavonoids, including neohesperidin, naringin, and neoeriocitrin. The study was carried out according to high-quality research standards to minimize bias. The effectiveness of this extract was also proved by the comparable results between this randomized, placebo-controlled clinical trial and a previous 6-month prospective study without placebo and short-term evidence conducted on the same extract [29].

The extract was effective in significantly reducing the LDL-C levels starting from the second month of intake, with a maximum effect after four months of use and a variation of LDL-C levels classification from “high level” (160–189 mg/dL) to “borderline high” (130–159 mg/dL). The LDL-C levels reduction was followed by a decrease of the TC and an increase of HDL-C.

The effect on HDL-C was modest and variable, as described in the scientific literature [2]. This finding aligns with the results reported by Toth et al., who observed an 8% increase in HDL-C levels in their clinical trial using the same extract [29]. Further consistency is provided by the systematic review conducted by Lamiquiz-Moneo et al., which found that eight out of twelve included studies that reported significant improvements in HDL-C levels, following bergamot supplementation [59]. However, the review also highlighted variability among studies, with some reporting no significant changes and others noting slight reductions in HDL-C levels, ranging from 1% to 6.5%. This variability may stem from the lack of standardization of active ingredients across studies, combined with the biological complexity of flavonoids, such as their bioavailability and biotransformation, all of which significantly influence their in vivo effects. Flavonoids are well-documented for their potent antioxidant and anti-inflammatory properties, which can enhance HDL functionality and contribute to overall cardiovascular health [60]. These effects have been particularly evident in dietary studies where they have been linked to cardiovascular benefits, especially in individuals with low baseline HDL-C levels—a known risk factor for cardiovascular disease (CVD) [61,62,63,64,65]. Although the observed increase in HDL-C was modest, its reproducibility, comparing this clinical trial with Toth et al., [29] suggests potential cardiovascular benefits, particularly for individuals at higher risk due to low HDL-C concentrations. These findings underscore the potential role of Bergavit as a dietary supplement in promoting cardiovascular health.

Interesting results were obtained for the ox-LDL levels and PON1 activity, confirming the well-known antioxidant efficacy of the bergamot flavonoids [65]. From literature data, for every 1% reduction in LDL-C, there is an approximate 1% reduction in CVD risk [12], and for every 1 mg increase in HDL-C, there is a 3–4% reduction in CVD risk [66]. The decrease of CVD risk is also associated with PON1 activity [67]. All totalling a ~20% reduction in CVD risk.

This study provides valuable insights as a randomized, placebo-controlled trial conducted with high methodological standards and using a standardized bergamot extract to ensure reproducibility. Significant improvements were observed in key lipid parameters over a relatively short timeframe, highlighting the potential of this intervention. This is the second clinical trial conducted using the same bergamot extract at the same dosage. By doing this, it was possible to validate the extract’s lipid-lowering effects in a larger population, providing further evidence of its potential benefits while ensuring consistency with established findings [29]. The absence of post-intervention follow-up and lack of monitored dietary intake for both the treatment and the placebo groups could represent a limitation and therefore will be examined in future research studies.

## 5. Conclusions

In conclusion, the oral intake of a characterized extract containing a standardized amount of flavonoids (150 mg/day) from *Citrus bergamia,* including neohesperidin, naringin, and neoeriocitrin, was significantly effective in reducing TC and LDL-C levels in the short and long term, starting from the second month of intake.

## Figures and Tables

**Table 1:** Demographic and baseline characteristics.

	Active (n = 30)	Placebo (n = 30)	*p* Value ^1^
Age (years)	50.9 ± 1.7	51.5 ± 1.6	0.8162
Sex			
Male	18 (60%)	16 (53%)	0.6054
Female	12 (40%)	14 (47%)	0.6054
Weight (Kg)	80.6 ± 1.8	80.8 ± 1.9	0.9268
BMI	27.8 ± 0.3	27.9 ± 0.4	0.8846
Blood pressure			
Systolic (mmHg)	123.1 ± 2.7	125.4 ± 2.8	0.5575
Diastolic (mmHg)	79.0 ± 1.4	79.8 ± 1.8	0.7268
LDL-C (mg/dL)	171.8 ± 1.6	171.3 ± 1.5	0.8190
HDL-C (mg/dL)	45.1 ± 2.0	49.4 ± 2.4	0.1642
TC (mg/dL)	243.0 ± 4.9	241.8 ± 2.8	0.8425
ox-LDL (ng/ml)	101.3 ± 3.7	103.5 ± 3.1	0.6526
TG (mg/dL)	131.1 ± 19.5	105.9 ± 6.8	0.2251
Glycemia (mg/dL)	99.4 ± 2.5	97.2 ± 2.3	0.5237
AST (U/L)	21.7 ± 1.7	22.5 ± 1.5	0.7367
ALT (U/L)	24.9 ± 2.6	26.6 ± 3.5	0.7081
GGT (U/L)	37.5 ± 7.2	33.4 ± 6.2	0.6662
HbA1c (%)	5.2 ± 0.1	5.2 ± 0.1	0.5120
PON1 (mU/mL)	90.0 ± 1.4	84.9 ± 2.2	0.0522
CRP (mg/L)	3.6 ± 1.1	3.8 ± 1.2	0.9041
CR (mg/dL)	1.01 ± 0.03	0.99 ± 0.03	0.6204

Continuous variables are presented as mean ± standard error, and categorical variables are shown as counts and percentages (in brackets). ^1^ Two-way, Student’s *t*-test for independent samples.

**Table 2:** Secondary endpoints. n.d. not measured. ** *p* < 0.01 (intragroup statistical analysis); # *p* < 0.05 (intergroup statistical analysis).

	Active (n = 30)				Placebo (n = 30)			
	M0	M2	M3	M4	M0	M2	M3	M4
Weight (Kg)	80.6 ± 1.8	81.1 ± 1.5	80.2 ± 1.7	80.1 ± 1.4	80.8 ± 1.9	81.1 ± 1.7	80.8 ± 1.5	81.0 ± 1.8
Blood pressure								
Systolic (mmHg)	123.1 ± 2.7	122.5 ± 2.3	119.8 ± 2.6	114.4 ± 1.8 **^,#^	125.4 ± 2.8	121.7 ± 2.8	120.1 ± 1.9	120.0 ± 2.2
Diastolic (mmHg)	79.0 ± 1.4	79.3 ± 1.1	78.4 ± 0.9	78.3 ± 0.9	79.8 ± 1.8	78.2 ± 1.7	77.6 ± 1.1	79.1 ± 1.1
TG (mg/dL)	131.1 ± 19.5	128.2 ± 14.6	111.6 ± 10.4	109.2 ± 12.5	105.9 ± 6.8	98.8 ± 6.8	101.3 ± 8.2	98.1 ± 6.2
Glycemia (mg/dL)	99.4 ± 2.5	n.d.	96.8 ± 1.7	96.7 ± 2.0	97.2 ± 2.3	n.d.	96.1 ± 2.2	97.5 ± 2.1
AST (U/L)	21.7 ± 1.7	n.d.	23.3 ± 1.8	24.8 ± 2.2	22.5 ± 1.5	n.d.	23.1 ± 1.5	21.8 ± 1.6
ALT (U/L)	24.9 ± 2.6	n.d.	26.7 ± 2.9	28.1 ± 3.2	26.6 ± 3.5	n.d.	26.9 ± 3.6	25.7 ± 3.3
GGT (U/L)	37.5 ± 7.2	n.d.	37.5 ± 5.5	35.4 ± 5.3	33.4 ± 6.2	n.d.	35.1 ± 6.7	33.0 ± 6.2
HbA1c (%)	5.2 ± 0.1	n.d.	5.2 ± 0.1	5.3 ± 0.1	5.2 ± 0.1	n.d.	5.2 ± 0.1	5.2 ± 0.1
CRP (mg/L)	3.6 ± 1.1	n.d.	1.9 ± 0.7	2.0 ± 0.5	3.8 ± 1.2	n.d.	2.4 ± 0.6	2.9 ± 0.6
CR (mg/dL)	1.01 ± 0.03	n.d.	0.88 ± 0.04	0.86 ± 0.04	0.99 ± 0.03	n.d.	0.87 ± 0.04	0.84 ± 0.04

## Data Availability

The data presented in this study can be obtained upon request from the corresponding author. As they are the property of the study sponsor, Bionap Srl (95032 Piano Tavola Belpasso, CT, Italy), the data are not publicly available.
